# Reply to Giansanti, D. Why Has Digital Contact Tracing Worked Differently in Different Countries? Comment on “Cao et al. The Impact of Digital Contact Tracing Apps Overuse on Prevention of COVID-19: A Normative Activation Model Perspective. *Life* 2022, *12*, 1371”

**DOI:** 10.3390/life12101593

**Published:** 2022-10-13

**Authors:** Junwei Cao, Dong Liu, Guihua Zhang, Meng Shang

**Affiliations:** 1 School of Business, Yangzhou University, Yangzhou 225127, China; 2 Department of Global Business, Yeungnam University, Gyeongsan 38541, Korea; 3 Department of Business, Yeungnam University, Gyeongsan 38541, Korea; 4 School of Flight, Anyang Institute of Technology, Anyang 455008, China

Thank you for your comments [1] for our Manuscript. We would like to discuss with you the question, “Why has digital contact tracing worked differently in different countries?” from the perspective of national disaster cultures.

Many studies in Western countries have shown that digital contact tracking APPs (DCTAs) cause a lot of inconvenience, leading to their effectiveness in preventing COVID-19, such as digital divide issues, diffusion issues, and data security issues [2,3,4]. In particular, one review noted that compared to Western DCTAs, the biggest problem with Chinese DCTAs (such as the Alipay Health Code app) is the difficulty in achieving an optimal balance between data protection standards and public health interests [3]. However, our study shows that the massive overuse of DCTA in China, while causing inconvenience, also helps to stimulate a sense of consequence and responsibility, in which people tend to ignore the inconvenience and consistently prevent COVID-19 [5]. I think it helps to understand such differences from the perspective of national disaster culture.

There are three main types of disaster management culture: state-oriented, individualistic, and fatalistic. In a state-oriented risk management culture, people believe that the direction of disasters is determined by both the environment and people, and that action must be taken to manage disasters. However, people do not know much about coping mechanisms, so they show a high level of trust in national disaster management authorities and have high expectations of the role of government. They believe that national-level involvement can be effective in managing disasters. They will trust and cooperate with the disaster management measures of the national disaster management authority and will be highly compliant with these measures [6,7]. In a culture of individualistic-centered disaster management, people believe that risk prevention is possible and that negative consequences should be reduced through action. However, they tend to have a high level of awareness of how they can respond to disasters, are not trusting of the role of government disaster management, and prefer to take action on their own to protect themselves [6,7]. In a fatalistic risk management culture, people will believe that disasters are forces of nature that cannot be denied, are unpredictable, and are inevitable. These people lack confidence in crisis resolution, and they expect the nation’s disaster management authorities to take action, but do not take nationally issued information about disasters seriously, and do not seriously cooperate with disaster management [6,7].

The national culture of disaster management in Chinese society is state oriented. Chinese people are highly subservient to the government’s instructions and arrangements to resolve their crisis. Chinese people have a high level of trust in the national disaster management authorities, and they believe that state involvement helps to manage disasters efficiently. As the Chinese government takes strong measures to prevent COVID-19, it inspires the public to cooperate with the government by creating a sense of collective responsibility and mission. Chinese people like to obtain knowledge and policies on epidemic prevention released by the government from social media, and also tend to express their views on responsibility and concerns about the epidemic through social media. Therefore, when the Chinese government overused DCTA nationwide, although it caused inconvenience to people, they were more inclined to sacrifice their own interests to cooperate with the state’s epidemic prevention when weighing the pros and cons in a state-oriented disaster response culture. Chinese also tend to show compassion for patients, blame those who caused the consequences and give appreciation to COVID-19 prevention heroes on social media [8,9]; in addition, with DCTAs’ tracking feature, the troublemakers who caused the spread of COVID-19 can be better located. Thus, although the Chinese perceived a lack of transparency in the operation of DCTAs and an unclear scope of data storage, they were also willing to overlook the potential loss of personal benefits from DCTAs [10] and cooperate with the national disaster authorities for effective use; ultimately, DCTAs have been shown to be extremely effective in China. Moreover, the sense of responsibility is very strong not only in China, but also in countries with Eastern cultures, such as South Korea, which has been very successful in preventing COVID-19. A study analyzed Korean posts on Twitter about COVID-19 prevention and found that the most content was about “attribution of responsibility” [11]. Korea is also a country where DCTAs are used on a large scale, and where the epidemic prevention department gives each place a phone number for people to register their tracks. If someone is infected with COVID-19, his or her track will likely be made public on the disease administration’s website.

In contrast, in some western countries, their disaster management culture may be centered on individualism. For example, in Europe, many British people refuse to use such apps because of privacy concerns [12], Irish people refuse to use it because they fear that tech companies or the government will use the app to monitor users even after the COVID-19 pandemic is over [13]. In the individualistic-centered disaster management culture, they are distrustful of government or company-led preventive measures. They are worried about their own interests and do not use DCTAs, and they prefer to face COVID-19 through their own perceptions rather than using DCTAs promoted by the relevant authorities or the government. Therefore, we can also infer that this part of the population can be individualistic in disaster management issues when they are capable of coping with COVID-19, and once they fail to cope, then they are likely to turn to a fatalistic disaster management culture, believing that COVID-19 is a force of nature, unpredictable and unavoidable, lacking confidence in the prevention of COVID-19, hoping to rely on the help of national disaster management authorities, but not taking national control measures, such as the strict use of DCTAs seriously.

Therefore, I think that future research can empirically study the effectiveness of DCTAs in different countries from the perspective of national disaster management culture, which will help each country to construct its own disaster management plan to cope with possible future international public health emergencies.

## Data Availability

The data presented in this study are available upon request from the corresponding author. The data are not publicly available for ethical reasons.
